# Role of targeted reassurance breast ultrasound in women with symptoms of a breast lump and normal clinical examination

**DOI:** 10.1186/bcr3280

**Published:** 2012-11-09

**Authors:** R Gallagher, H Burrell, E Cornford

**Affiliations:** 1 Nottingham Breast Institute, Nottingham, UK

## Introduction

Ultrasound has a well-established role in the triple assessment of breast lumps. The purpose of this study was to review the value of targeted breast ultrasound in women presenting with symptoms of a breast lump but normal clinical examination.

## Methods

The records of all patients presenting to the symptomatic clinic with a breast lump but with a normal clinical examination over a 6-month period were reviewed. In our institution all such patients undergo targeted ultrasound of the symptomatic area within the breast; and mammography if aged 40 years or over. The results of the ultrasound examination, mammography and histology from any needle biopsy were reviewed.

## Results

Seven hundred and ninety-nine women were included (mean age = 39, age range 15 to 84). One hundred and two (13%) had an abnormality detected on ultrasound, 92 (90%) of which were at the same site as the patient's symptoms. Thirty-two patients (4% of the study group) had solid lumps and 70 (9%) had cysts. Five cancers were detected, four of which were at the site of the patient's symptoms and one of which was an incidental area of DCIS visible on mammography only. Three of the four patients with invasive cancer had a normal mammogram.

## Conclusion

Ultrasound is a useful diagnostic tool in patients who present with a breast lump, but who have a normal clinical examination. In this study a small number of cancers were detected. The value of a normal or benign ultrasound in reassuring both patient and clinician is more difficult to quantify.

